# Does caffeine consumption affect laparoscopic skills in a motion tracking analysis? A prospective, randomized, blinded crossover trial

**DOI:** 10.1007/s00464-021-08783-6

**Published:** 2021-11-15

**Authors:** Felix von Bechtolsheim, Florian Oehme, Michael Maruschke, Sofia Schmidt, Alfred Schneider, Jürgen Weitz, Marius Distler, Sebastian Bodenstedt, Isabel Funke, Stefanie Speidel, Soeren Torge Mees

**Affiliations:** 1 Department for Visceral, Thoracic and Vascular Surgery at the University Hospital Carl Gustav Carus, Technische Universität Dresden, Dresden, Germany; 2 Centre for Tactile Internet with Human-in-the-Loop, Technische Universität Dresden, Dresden, Germany; 3 Division of Translational Surgical Oncology, National Center for Tumor Diseases, Partner Site Dresden, Fetscherstraße 74, 01307 Dresden, Germany; 4 Department of General, Visceral and Thoracic Surgery, Städtisches Klinikum, Friedrichstraße 41, 01067 Dresden, Germany

**Keywords:** Caffeine, Coffee, Laparoscopic skill analysis, Laparoscopic motion analysis, Proficiency-based learning

## Abstract

**Background:**

Coffee can increase vigilance and performance, especially during sleep deprivation. The hypothetical downside of caffeine in the surgical field is the potential interaction with the ergonomics of movement and the central nervous system. The objective of this trial was to investigate the influence of caffeine on laparoscopic performance.

**Methods:**

Fifty laparoscopic novices participated in this prospective randomized, blinded crossover trial and were trained in a modified FLS curriculum until reaching a predefined proficiency. Subsequently, all participants performed four laparoscopic tasks twice, once after consumption of a placebo and once after a caffeinated (200 mg) beverage.

Comparative analysis was performed between the cohorts. Primary endpoint analysis included task time, task errors, OSATS score and a performance analysis with an instrument motion analysis (IMA) system.

**Results:**

Fifty participants completed the study. Sixty-eight percent of participants drank coffee daily. The time to completion for each task was comparable between the *caffeine* and *placebo* cohorts for PEG transfer (119 s vs 121 s; *p* = 0.73), precise cutting (157 s vs 163 s; *p* = 0.74), gallbladder resection (190 s vs 173 s; *p* = 0.6) and surgical knot (171 s vs 189 s; *p* = 0.68). The instrument motion analysis showed no significant differences between the caffeine and placebo groups in any parameters: instrument volume, path length, idle, velocity, acceleration, and instrument out of view. Additionally, OSATS scores did not differ between groups, regardless of task. Major errors occurred similarly in both groups, except for one error criteria during the circle cutting task, which occurred significantly more often in the caffeine group (34% vs. 16%, *p* < 0.05).

**Conclusion:**

The objective IMA and performance scores of laparoscopic skills revealed that caffeine consumption does not enhance or impair the overall laparoscopic performance of surgical novices. The occurrence of major errors is not conclusive but could be negatively influenced in part by caffeine intake.

**Supplementary Information:**

The online version contains supplementary material available at 10.1007/s00464-021-08783-6.

Surgery is a demanding profession, both physically and cognitively. It is characterized by long working days, high numbers of on-call duties per month and a higher subjective work overload [1, 2]. Therefore, it seems not particularly surprising that surgeons are prone to higher coffee consumption.

Giesinger et al. showed in 2015 that orthopedic surgeons purchased the most coffee during hospital working hours, followed by radiologists and general surgeons [3]. The most common reasons for caffeine usage among surgeons were to reduce fatigue (54.3%), work night shifts (32.2%) and excessive work hours (31.7%). The past-week prevalence for coffee, caffeinated drinks and caffeine tablets was 50.5%, 6.1% and 3.8%, respectively. A total of 623 surgeons (70.16%) used caffeinated substances with the particular purpose of enhancing cognitive capabilities at least once in their lifetime [4]. A survey among German surgeons revealed that 48% of surgeons drank more than 3 cups of coffee per day, whereas only 9% did not consume coffee at all [5].

The popularity of caffeine is largely due to the many effects attributed to it. The major pharmacological effector in coffee is caffeine. Caffeine can be found in more than 60 different plants, including cocoa and tea [6]. Caffeine is distributed in all body tissues and can cross the blood–brain barrier [7].

Among the many effects of caffeine within the central nervous system, it is claimed that it increases vigilance and performance, especially during limited sleep deprivation [8]. The intake of caffeine proved to elevate the alertness of participants, even after a prolonged wakefulness of more than 48 h [9]. However, caffeine might also cause a certain dependency and, therefore, partially result in deteriorating effects, as James and Rogers discussed. They claimed that caffeine withdrawal actually degrades mood and performance in most coffee consumers. Consequently, caffeine consumption does not improve mood and performance beyond an individual baseline, but restores mood and performance after a low caused by caffeine-deficiency [10].

In addition to the fatigue counteracting effect, many people also use caffeine for cognitive enhancement [11]. Hence, it is not particularly surprising that the caffeinated hot drink coffee is particularly popular among surgeons.

The hypothetical downside of caffeine in the surgical field is the potential interaction with the ergonomics of movement, which is essential for delivering high-quality surgery. Caffeine can cause acute blood pressure elevation, increased metabolic rate and diuresis [6, 12]. Furthermore, it stimulates locomotor activity [13–15]. This leads to the apprehension that caffeine potentially could influence surgical performance. As a result, Fargen et al. recommended for neurosurgeons to avoid caffeine to improve the surgical dexterity [16].

However, the evidence for the impact of caffeine on surgical skills is little and inconclusive [17]. But most research regarding this topic focuses on specialities like ophthalmological and oto-surgical microsurgery, where tremor prevention is highly favorable. The research about any beneficial or disadvantageous effects of caffeine on laparoscopic skills, on the other hand is underrepresented.

Therefore, the primary objective of this prospective and randomized crossover trial was to investigate the extent to which caffeine influences the laparoscopic performance of young naive surgeons during the execution of fundamentals in laparoscopic surgery (FLS) tasks.

## Materials and methods

This article was written in accordance with the CONSORT statement [18]. The trial was conducted as a prospective, randomized controlled, blinded crossover study. The experimental protocol of the study was approved by the local ethics committee of the TU Dresden (Decision Number EK 416092015). All experimental methods were carried out in accordance with relevant guidelines.

### Participants

A total of 50 medical students participated in this trial. All participants consented to participation and the consumption of caffeine after thorough presentation of information by the principal investigators. All participants took part in an elective course for the training of minimally invasive surgery. The training was conducted until all students reached a predefined proficiency level. The curriculum was based on a modified FLS curriculum, and the detailed curriculum and thresholds for the proficiency level have been described previously [19, 20]. Surgical novices were deliberately chosen, because the training to a predefined proficiency ensured a high comparability between all participants. Moreover, the missing experience and training of young surgeons might make them more susceptible to influencing factors, and thus show a potential effect of caffeine more clearly.

Participants answered a questionnaire, specifically created for this study, at the beginning of the teaching course investigating basic participant information (e.g., age, sex, study semester, handedness) and personal caffeine consumption habits (e.g., which kind of caffeine consumption, estimated amount of caffeine consumption per day) and the subjectively felt effect of caffeine consumption (e.g., reduced tiredness, enhancement of concentration, positive effect on stress perception).

### Testing

All participants were asked to avoid drinking beverages containing caffeine for a minimum of 4 h prior to the laparoscopic skill analysis. This caffeine fasting period was chosen based on the literature stating that the half-life of caffeine in healthy adults is mostly between 4 and 6 h [6, 7, 10, 13, 21].

All participants received either a placebo or a caffeinated (200 mg caffeine) beverage. Both were dissolved as a powder in decaffeinated coffee to equalize the taste of both applied beverages. The dose of caffeine was chosen as an equivalence of approximately 2 cups of coffee. Literature research revealed that 1 cup of coffee contains between 70 and 130 mg of caffeine [22, 23]. Investigators interacting with the participants were blinded regarding the caffeine or placebo application.

Each participant was tested twice, once receiving caffeine and once receiving a placebo. The order of caffeine or placebo application was randomized. Between each test, there were at least 24 h for each participant to reduce learning effects.

A second questionnaire, also specifically created for this study, had to be filled after receiving the placebo or the caffeinated beverage and before the skill analysis, respectively. This questionnaire included questions about the sleeping status of the last night as well as coffee intake (as units 250 ml), sports, and study intensity for that specific day. Additionally, the questionnaire asked the participants whether they would feel more relaxed, powerful, concentrated, happier, restless, or uncomfortable after drinking the beverage containing caffeine or placebo. Additionally, participants were asked to identify whether they received caffeine or placebo. A second questionnaire, answered only at one of the testing occasions, asked for usual caffeine intake, personal value of caffeinated drinks and smoking habits.

All participants had to wait 30 min before continuing with the analysis of the laparoscopic performance, as the peak plasma concentration of caffeine is reached within 15–20 min after oral intake [6]. Before and 30 min after the intake of caffeine or placebo, the vital parameters (systolic blood pressure, diastolic blood pressure and heart rate) of each participant were measured.

Eventually, the participants had to perform four different laparoscopic tasks, which were described in detail previously [20]. The laparoscopic tasks were the same, and they were trained up to proficiency: PEG transfer, circle cutting, gallbladder and laparoscopic suture.

### Instrument motion analysis (IMA)

The experimental setup to test the participants consisted of a box trainer (Laparo Aspire®), an optical tracking system (NDI Polaris®) and laparoscopic instruments (forceps, Overholt, scissor, needle holder) with marker spheres attached to them. The tracking system consists of two infrared cameras, which are able to locate these marker spheres. After calibrating the relative position of the handle to the instrument tip, the system can track the motion of the instrument tip in space. Using different patterns of marker spheres on each instrument helps to differentiate the motion of different instruments.

The performance data from the motion tracking system were obtained for both instruments as well as separately for only the left or the right instrument. Variables included percentage of task time the instrument was out of the endoscopic view, percentage of task time the instrument was idle, pathway of the instrument, velocity of the instrument, acceleration of the instrument, and volume of motion. Here, the volume of motion corresponds to a cube whose sides are defined by the respective widest motion of the laparoscopic instruments in the *x*-, *y*- and *z*-axis. Therefore, this parameter represents a three-dimensional space defined by the path of the laparoscopic instrument.

### Performance rating

All videos were recorded and assigned for subjective performance ratings using a modified OSATS score (Supplementary Material). The OSATS score was modified to fit the experimental setting. Participants were rated using a Likert scale from 1 to 5 on four different criteria: depth perception, efficiency, bimanual handling and tissue handling. The maximum OSATS score was 20, whereas the minimum was 4. Furthermore, major errors were defined for each task, and the occurrence of such errors was recorded.

All videos were rated by a specifically trained rater with experience in the FLS curriculum. The videos were presented to the rater in random order without any information containing data about the participant or about the influence of caffeine or placebo.

### Statistical analysis

Statistical analysis was carried out using SPSS version 26 (IBM Corp, Armonk NY, USA). The normality of continuous data was tested with the Kolmogorov–Smirnov test and by inspecting the frequency distributions. The participant characteristics are represented either as medians and interquartile ranges (IQRs) for continuous variables or as distributions of frequencies. The crossover analysis was chosen depending on the data characteristics (paired Student’s *t*-test, McNemar’s test, Wilcoxon rank test). There were no missing values for the primary analyses. The threshold for the level of significance was defined as *p* ≤ 0.05.

## Results

### Participants

The mean age of the participants was 23 years, and 31 (62%) students were female. Right handedness was predominant, with 44 (88%) participants being right-handed. Most students (78%) were in their fourth year of study. Only eight participants (16%) were smokers on a regular basis (Table 1).

**Table 1:** Basic participant characteristics, caffeine intake habits and basic questionnaire results

Total number of participants	50
Age [years] (IQR)	23 (22.75–24)
Sex [*n* (%)]	
Female	31 (62)
Male	19 (38)
Handedness [*n* (%)]	
Right	44 (88)
Left	6 (12)
Year of attendance [*n* (%)]	
Third year students	4 (8)
Fourth year students	39 (78)
Fifth year students	7 (14)
Daily coffee intake [*n* (%)]	34 (68)
Daily (other) caffeinated drinks [*n* (%)]	25 (50)
Only daily (other) caffeinated drinks [*n* (%)]	2 (4)
Total units (250 ml) coffee per day	1.5 (0.8–2.5)
Total units (250 ml) coffee per week	10 (1.9–14.3)
Do you need Coffee to …. [*n* (%)]	
… start the day?	17 (34)
… regain energy and concentration?	32 (64)
… relax?	22 (44)
Smoker [*n* (%)]	8 (16)

### Caffeine consumption

The majority of participants (68%) stated that they drink coffee daily (Table 1). Half of the participants (50%) indicated the consumption of other caffeinated drinks. The overlap between those consuming coffee and caffeinated drinks was 23 students, and only two (4%) drank only caffeinated drinks on a daily basis. Among the participants with daily coffee consumption, the mean intake was 1.5 units (375 ml) of coffee per day and 10 units per week. Participants were asked if they agreed with certain statements related to coffee consumption. Seventeen participants (34%) agreed with the statement that they need coffee to start the day, whereas 32 (64%) stated that coffee would help them regain energy and concentration. Twenty-two (44%) participants needed coffee to relax.

### Pre-test questionnaire

Participants felt unaltered after drinking the caffeinated beverage or the placebo in terms of relaxation, power, concentration, happiness, or restlessness. On the other hand, significantly more participants felt uncomfortable after consuming caffeine compared to those participants drinking the placebo (34 vs. 8%, *p* < 0.01). In both groups, placebo and caffeine, the pre-test sleeping hours, pre-test sport activity and lecture sessions were similar. Additionally, coffee intake prior to the 4-h caffeine restriction period before the test did not significantly differ in either group. Interestingly, 66% of participants correctly identified the beverage containing caffeine, whereas only 52% of participants correctly identified the application of a placebo (*p* < 0.01) (Table 2).

**Table 2:** Vital parameters and pre-test questionnaire

	Placebo group	Caffeine group	*p* Value
Blood pressure [mmHg] (IQR)^a^			
Systolic pressure before intake	115 (110–120)	118 (115–125)	0.12
Systolic pressure after intake	113.5 (105–123.8)	120 (111.3–125)	**< 0.05**
Diastolic pressure before intake	70 (65–75)	71.5 (65–79.5)	0.19
Diastolic pressure after intake	70 (65–75)	75 (70–80)	0.98
Heart rate [*N*] (IQR)^a^			
Before intake	72 (65–83)	74 (68.5–84)	0.14
After intake	73 (65–80)	72 (64–80)	0.64
Drinking the beverage made me feel …. [*n* (%)]^b^			
… more relaxed	13 (26)	10 (20)	0.58
… more powerful	11 (22)	19 (38)	0.15
… more concentrated	7 (14)	8 (16)	1
… happier	13 (26)	8 (16)	0.23
… restless	14 (28)	24 (49)	0.09
… uncomfortable	4 (8)	17 (34)	**< 0.01**
Pre-test sleeping hours [h] (IQR)^a^	7 (6–8)	7 (6–8)	0.43
Pre-test coffee intake [units] (IQR)^a^	0 (0–1)	1 (0–1)	0.69
Pre-test sports [min] (IQR)^a^	20 (10–30)	20 (12.5–27.5)	0.45
Pre-test lecture sessions [h] (IQR)^a^	1.5 (1.5–3–5)	1.5 (1.5–2)	0.68
Correct identification of caffeine or placebo [n] (%)^c^	26 (52)	33 (66)	**< 0.01**

^a^Paired students *t*-test

^b^McNemar test

^c^χ^2^-test

Significant values are marked bold

### Vital parameters

There was no significant difference in systolic blood pressure before intake of caffeine or placebo, with 118 and 115 mmHg (*p* = 0.12), respectively. Likewise, the diastolic blood pressure did not differ between the groups before drinking the caffeinated or the placebo beverage. The blood pressure after intake of caffeine was significantly higher in the caffeinated group (120 vs. 113.5 mmHg, *p* < 0.05). The diastolic blood pressure after intake of caffeine or placebo revealed no significant differences between the groups. The heart rate measured before (74 vs. 72 bpm, *p* = 0.14) and after (75 vs. 70 bpm, *p* = 0.64) consuming the beverage did not differ significantly between groups (Table 2).

### Time

After consuming caffeine, the participants completed PEG transfer, circle cutting, gallbladder resection and surgical knot surgery in 119 s, 157.1 s, 189.9 s and 171.3 s, respectively. In the placebo group, participants needed 120.7, 163, 172.9 and 188.8 s, respectively. There were no significant differences in the task time between the two groups (Table 3).

**Table 3:** Completion time per task

Completion time per task [s] (IQR)	Placebo	Caffeine	*p* Value
PEG transfer	120.7 (105.8–136.6)	119 (106.4–143.5)	0.73
Circle cutting	163 (127.1–190.8)	157.1 (130.8–184.2)	0.74
Gallbladder resection	172.9 (141–231.9)	189.9 (154.9–246.1)	0.6
Surgical knot	188.8 (133.2–229)	171.3 (135.3–244.6)	0.68

### Instrument motion analysis

The volume of both instruments did not differ significantly between the caffeine and placebo groups on all tasks (Table 4). In the gallbladder resection and the surgical knot task, both the caffeine and the placebo groups showed higher volumes than the PEG transfer and circle cutting tasks. There were also no differences between the groups regarding the individual left and right instrument volume in any of the tasks. In most of the tasks, both groups showed a higher instrument volume on the left side. In contrast, a longer path length was observed for the right instrument on all tasks, except the surgical knot task. Nevertheless, the path length showed no significant differences between the caffeine and placebo groups on any of the tasks. Similarly, the idle instrument was comparable between the two groups. Regarding the velocity of instruments, the right instrument tended to be faster in almost all tasks, except for the placebo group performing gallbladder resection. Overall, there were no significant differences between the caffeine and placebo groups regarding either the left or the right instrument velocity. Interestingly, in the surgical knot task, the right instrument acceleration was multiplied compared to the left side. However, again, both groups were comparable with the other tasks. The left instrument showed a higher tendency to be out of view, except for the PEG transfer task. This observation could be made for both groups, and therefore, no significant difference could be seen.

**Table 4:** Instrument motion analysis parameters

Motion parameter	PEG transfer			Circle cutting			Gallbladder resection			Surgical knot		
	Placebo (25–75 IQR)	Caffeine (25–75 IQR)	*p*	Placebo (25–75 IQR)	Caffeine (25–75 IQR)	*p*	Placebo (25–75 IQR)	Caffeine (25–75 IQR)	*p*	Placebo (25–75 IQR)	Caffeine (25–75 IQR)	*p*
Both instruments, volume (cm^3^)	1138.7 (938–1399.3)	1165.5 (990.9–1343.6)	0.38	1105.4 (856.5–1844.4)	1268.3 (971.5–1765.2)	0.85	1839.1 (1238.5–2490.3)	1703.1 (1309.7–2395.8)	0.64	1702.8 (1432.6–2169.9)	1746 (1248.9–2105.4)	0.52
Left instrument, volume (cm^3^)	600.7 (458.3–754.5)	584 (451.5–751.9)	0.42	816.6 (636–1232.6)	969.6 (653–1381.8)	0.6	1097.9 (735.4–1647.4)	1096.2 (743.6–1861.6)	0.61	1126.9 (812.4–1711.7)	1043.7 (679.1–1636.4)	0.14
Right instrument, volume (cm^3^)	552.5 (418.4–732.1)	563.5 (461.6–704.4)	0.54	656.9 (434.9–892.69)	637.7 (475.8–1036.3)	0.5	1058.4 (844.4–1262.9)	1124 (701–1549.5)	0.98	770.7 (507.9–1043.2)	660.3 (448.9–921)	0.69
Left instrument, path length (cm)	392 (329.3–463.8)	413.1 (333.6–539.4)	0.3	384.1 (284.1–520.9)	387.2 (286–528.3)	0.98	421.8 (328.1–524.9)	421 (296.4–553.5)	0.79	369.6 (294.6–580.9)	373.8 (326.8–516.6)	0.73
Right instrument, Path length (cm)	398.5 (333.6–471.7)	414.9 (354.6–498.7)	0.5	456 (333.3–608.8)	511.4 (358.8–628.7)	0.73	452.9 (327.5–569)	464.9 (345–580.5)	0.49	277 (137.2–420.8)	304.5 (209–410.6)	0.26
Left instrument, idle (%)	58.1 (53.5–61.8)	57.1 (52.3–61.1)	0.81	58 (48.175.2)	61 (51.7–73.3)	0.54	66.1 (57.5–75.5)	64.7 (54.6–71.2)	0.15	67.7 (60.8–73)	68.4 (61.9–73.4)	0.58
Right instrument, idle (%)	54.6 (44.1–62.2)	55 (46.9–62.6)	0.8	55.4 (46.8–65)	58.3 (46.8–65)	0.44	68.6 (61.8–71.8)	65.5 (59.3–73.8)	0.12	60.9 (55.3–67.1)	62.1 (56.7–66.1)	0.53
Left instrument, mean velocity (mm/s)	33.8 (30.8–36.6)	33.8 (30.7–38.9)	0.11	31 (18.8–37.7)	30 (20.5–36.1)	0.35	28.5 (22.8–31.1)	26.3 (23.9–31.1)	0.24	26.9 (22.9–30.1)	27 (23.3–30.5)	0.46
Right instrument, mean velocity (mm/s)	34.9 (29.9–43.1)	35 (30–41.4)	0.76	33.4 (27.4–41.2)	32.5 (27.8–41)	0.6	28.1 (24.1–31.6)	27.9 (24.3–33.7)	0.13	30.8 (26.5–35.5)	31.3 (28.8–35.4)	0.13
Left instrument, acceleration (mm/s^2^)	1.98 (1.22–2.8)	2.1 (1–3.3)	0.99	2.9 (1.9–4.8)	2.8 (1.7–4.4)	0.27	2.5 (1.6–4.6)	3.5 (2.2–6.1)	0.73	4 (2.1–7)	3.8 (2.1–7.1)	0.89
Right instrument, acceleration (mm/s^2^)	2.6 (1.2–4.2)	2 (1–4.4)	0.33	2.7 (1.8–5.5)	2.9 (1.9–5.5)	0.22	2.7 (1.3–4)	3.6 (1.8–5.3)	0.38	14 (9.9–19.3)	12.7 (7.3–18.6)	0.94
Left instrument, out of view (%)	0.4 (0–1.3)	0.3 (0–1.4)	0.54	2.9 (1–9)	3.7 (0.9–9.9)	0.89	2.9 (1–12.5)	6.7 (1.4–16.8)	0.15	2.6 (1.4–5.5)	2.3 (1–4.1)	0.36
Right instrument, out of view (%)	0.7 (0–3.5)	0.5 (0–2.1)	0.55	1 (0–6.1)	1.5 (0.1–4.6)	0.41	2.4 (1–6.1)	3.2 (1–8.3)	0.09	0.5 (0–3.1)	0.3 (0–1.6)	0.21

### OSATS score

**Graph 1:**
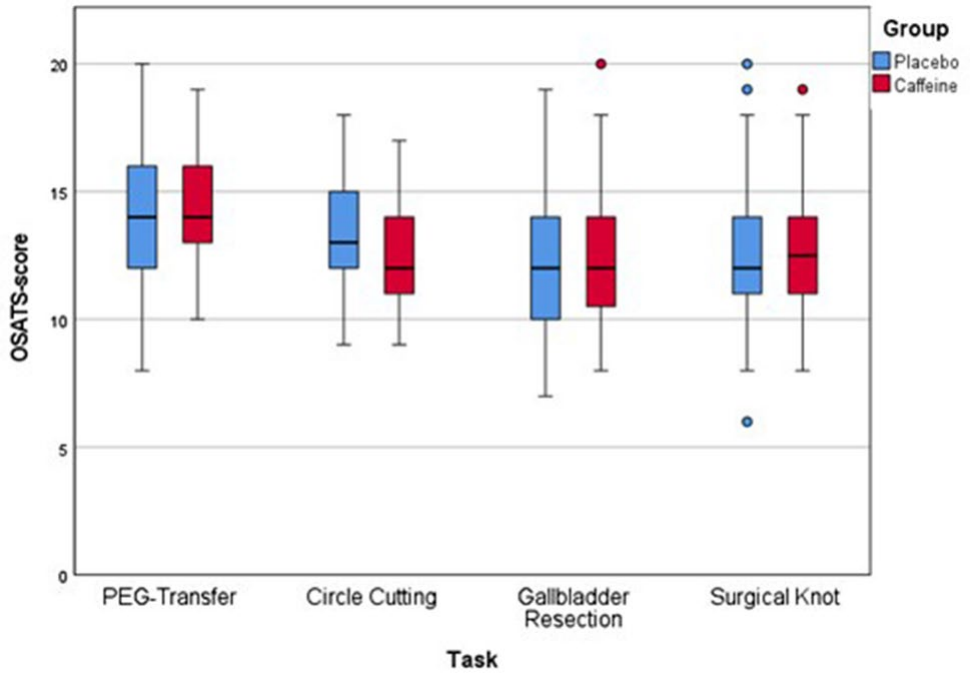
Box-plots of modified OSATS scores for all tasks

Both the caffeine and the placebo groups scored the highest OSATS of 14 in the PEG transfer task (Table 5; Graph 1). The caffeine group scored an OSATS of 13.0, 12.0 and 12.0 in the circle cutting, gallbladder resection and surgical knot tasks, respectively. However, the placebo group reached a median of 12.0, 12.0 and 12.5 in the same tasks, respectively. Subsequently, the comparable OSATS results on all tasks for both groups showed no significant difference.

**Table 5:** Modified OSATS score for all tasks

OSATS	Placebo		Caffeine		*p* value
	*n* (%)	Median (IQR)	*n* (%)	Median (IQR)	
PEG transfer	50 (100)	14.0 (12.0–16.0)	50 (100)	14.0 (13.0–16.0)	0.598
Circle cutting	50 (100)	13.0 (12.0–15.0)	50 (100)	12.0 (11.0–14.0)	0.122
Gallbladder resection	49 (98)	12.0 (10.0–14.0)	40 (80)	12.0 (10.25–14.0)	0.576
Surgical knot	50 (100)	12.0 (11.0–14.25)	50 (100)	12.50 (11.0–14.0)	0.987

### Major errors

Regarding PEG transfer, two major errors were recorded (Table 6). Both groups dropped more PEGs within than outside the view, but there were no significant differences between the caffeine and placebo groups in either error category (*p* = 0.791 and *p* = 1.0). There were also no significant differences regarding the error of cutting outside the marked line during the circle cutting task (*p* = 0.5), but significantly more participants in the caffeine group (caffeine: 34% vs. placebo: 16%, *p* = 0.049) dislocated the pad with the circle drawn on it from the mount. Gallbladder injury occurred relatively often in both groups. Nevertheless, the incidence of this error was comparable in both groups (*p* = 0.263). The percentage of participants cutting outside the marked line on the gallbladder was also similar in both groups (*p* = 0.687). Regarding the surgical knot task, both groups performed similarly in tightening all three knots (*p* = 0.581), inaccurate stitching (*p* = 1.0) and the occurrence of multiple stitches (*p* = 0.804).

**Table 6:** Major errors for all tasks

Major errors		Placebo		Caffeine		*p* Value
Task	Error definition	*n*	%	*n*	%	
PEG transfer	PEG drop within view	13	26	15	30	0.791
	PEG drop outside view	2	4	2	4	1.000
Circle cutting	Cut outside the line	1	2	3	6	0.500
	Dislocation of tissue	8	16	17	34	0.049
Gallbladder resection	Injury of gallbladder	22	44	28	56	0.263
	Cut outside the line	6	12	4	8	0.687
Surgical knot	All 3 knots tight	43	86	40	80	0.581
	Inaccurate stitching	5	10	4	8	1.000
	Multiple stitches	15	30	13	26	0.804

### Self-assessment: regular vs. occasional coffee consumption

Furthermore, the same variables were tested for significant differences between participants who stated to regularly drink coffee or caffeine beverages and participants who did not. Here, caffeine intake did not result in any significant differences for any motion variable (data not shown). In line with these findings, no systematic differences in terms of OSATS were seen between these participants. Even though a significantly higher number of participants reported feeling uncomfortable after drinking the caffeine beverage, these participants did not show significant differences in laparoscopic performance regarding OSATS, performance time or motion parameters.

## Discussion

The primary objective of this trial was to analyze the potential effects of caffeine on laparoscopic skills and motion ergonomics.

First, we found the time of completion to be comparable between both groups for all tasks without any systematic trend between the groups. Therefore, we assume that task completion efficiency is neither negatively nor positively influenced by caffeine consumption. This contrasts with a crossover trial from Quan et al., who found that coffee had a negative effect on task completion time in a virtual reality-simulated laparoscopy trial [24]. Quan argues that coffee has a negative effect on the motor component and consequently increases task completion time. However, participants were laparoscopic novice surgeons with no pre-task training, which might have influenced the results substantially. Hence, Quan et al. concluded that these results must be interpreted critically, and further trials with more experienced participants are recommended. With our train-to-proficiency approach, we believe that our results have far better transferability. The novel aspect of this trial was laparoscopic IMA mounted to a real box. This IMA provides valid and objective parameters that are easily comparable between participants. This allows for a deeper understanding of potential influences (e.g., caffeine) on surgical motion ergonomy and surgical performance. To date, only Kowalewski et al. used a similar system for laparoscopic skill analysis and proved validity and reliability [25].

The primary hypothesis was that caffeine consumption would alter the laparoscopic skills of surgeons. Interestingly, significant differences were not seen between the groups in terms of the IMA. The overall efficiency of motion, as indicated by the parameters of volume, path length, velocity, acceleration and idle, was not improved or diminished by caffeine consumption. Therefore, it can be assumed that basic laparoscopic psychomotor skills are neither decreased nor enhanced by caffeine.

Although research regarding tremor and its possible effect on surgical performance is limited, some authors recommend caffeine abstinence to avoid a worsening of a tremor [16, 17]. Our data, in particular the parameters “instrument idle” and “instrument path length”, do not suggest a difference due to a tremor in the caffeine group. A tremor would be expected to increase both parameters. In contrast, our system measured the instrument handles and might have missed a slight tremor, which can increase at the instrument tips due to the point of leverage depending on the depth of insertion in the laparoscopic trocars.

Whereas our research is based on resting participants, Aggarwal et al. first showed that performance in laparoscopic surgery was significantly worse if participants were sleep-deprived compared to their resting performance. However, the consumption of caffeine after 24 h of sleep deprivation restored the participants’ laparoscopic performance to their resting baseline performance. There was one exception: there was no difference in the number of errors produced by participants being sleep-deprived and after receiving caffeine [26]. Nonetheless, Aggarwal et al. basically investigated the influence of two variables: sleep deprivation and caffeine.

In addition, we assessed the laparoscopic performance employing the OSATS scores. The subjective rater analysis supported the assumption derived from the objective IMA: the OSATS scores between both groups were comparable on all tasks. Both groups achieved the best OSATS scores in the PEG transfer compared to the other tasks. This observation is most likely due to the simplicity and therefore decreased difficulty of the latter task.

There were mostly no significant differences in the occurrence of major errors during the tasks between the groups. Nevertheless, after caffeine consumption, participants tended to perform worse in six out of nine major error criteria. Only one error criterion showed significant differences: in 34% of cases, significantly more participants dislocated the pad with the circle drawn on it from its mount after caffeine consumption. This might indicate a higher force input or rougher tissue handling of participants in the caffeine group. However, this force exacerbation was not seen in other tasks, such as stitching or gallbladder resection. In summary, the results show no differences between the groups, with exception of one error criterion, the dislocation of tissue during the circle cutting task. Caffeine could possibly affect force exertion, but the data available here are not sufficient to draw a definitive conclusion in this regard.

Furthermore, our subgroup analysis did not show any differences in laparoscopic performance between participants who were used or not used to caffeine consumption. This observation might contradict a potential deteriorating effect of caffeine withdrawal for participants being used to it, as claimed by James and Rogers [10]. Our results also do not support the hypothesis of a direct negative effect of caffeine on surgical performance, as discussed by Urso-Baiarda et al. [21].

### Strengths and limitations

In this study, we decided to compare students without or with very little knowledge and practical skills regarding laparoscopic surgery. Surgical novices are more prone to influencing factors, and thus caffeine might have revealed an influence more easily. Moreover, the previous training until reaching a predefined proficiency level before undertaking the study ensured high comparability between the participants. It remains speculative whether trained surgeons are more or less susceptible to potential caffeine effects. The administration of 200 mg caffeine is realistic as our questionnaire showed. Regular coffee drinkers consumed an average 1.5 units (375 ml) of coffee daily, containing approximately a total of 175–325 mg of caffeine depending on the type of coffee [22]. Nevertheless, the daily coffee consumption can only be an approximation for the actual caffeine consumption, since the caffeine dose depends on the type of coffee (e.g., espresso, bean coffee, instant coffee, etc.) [22]. Furthermore, the study design might not have accounted for a potential withdrawal effect of caffeine. A longer period of caffeine abstinence might have increased the withdrawal effect of caffeine for participants with regular caffeine consumption.

Another limitation of our study is the restriction to only one rater for the video analysis and OSATS scoring. This can compromise the validity of our finding, due to the subjectivity of a single rater. On the other hand, this limitation of our research also shows the potential of automated and objectified surgical skill analysis, which could significantly contribute to the simplification of similar research, if no or less raters are necessary. Regarding the usage of the novel IMA tool, the parameters analyzed by us were selected based on their understandability and meaningfulness. Hence, surgeons should be able to understand and specifically work on these parameters. Furthermore, this motion analysis allows for a more detailed, subjective, and automated statement regarding surgical performance. Even though similar motion analysis is implemented in many laparoscopic virtual reality simulators, our system’s advantage is the possibility to reproduce the usage of real laparoscopic instruments with realistic instrument handling. In addition, the system’s versatility hypothetically allows it to be used in more complex scenarios, such as wet-lab operations.

## Conclusion

Our study revealed neither adverse nor beneficial effects of caffeine consumption nor effects of short-term caffeine withdrawal on laparoscopic surgical skills using a novel motion tracking skill analysis. These data enable far deeper insight into the relationship between laparoscopic movement economics and a potential influencing variable, such as caffeine. Our findings were strengthened by the fact that neither task completion time nor OSATS scores differed between participants after receiving caffeine. The occurrence of major errors also showed mostly no differences with exception of one error criterion, which occurred significantly less in the placebo group. Therefore, a potential adverse effect of caffeine can not be excluded and should be investigated in further research.

## Supplementary Information

Below is the link to the electronic supplementary material.

Supplementary file 1 (DOCX 35 kb)
