# Supplementary material for: Does caffeine consumption affect laparoscopic skills in a motion tracking analysis? A prospective, randomized, blinded crossover trial
Source: Surg Endosc. 2021 Nov 15;36(6):4359–68. doi: 10.1007/s00464-021-08783-6 (PMC9085661; doi:10.1007/s00464-021-08783-6)
Supplement: Supplementary file 1 — (DOCX 35 kb) [file 464_2021_8783_MOESM1_ESM.docx]

Supplementary Material

***Annotation Guideline - PEG-Transfer***

1. Depth Perception

| 1 | 2 | 3 | 4 | 5 |
| --- | --- | --- | --- | --- |
| - Instrument with slow movement toward PEG - Missing the object multiple times and multiple attempts - >2 attempts to transfer the object from one grasper to the other - Slow placement of object on PEG |  | - Instrument moves toward the PEG at a moderate speed and does not miss it - Transfer works at moderate speed - Set down possible without problems |  | - Instrument moves precisely, quickly and without lapse - The transfer is efficient and fast |

1. Efficiency

| 1 | 2 | 3 | 4 | 5 |
| --- | --- | --- | --- | --- |
| - the pick-up of the first object is unnecessarily delayed - the second instrument for transfer must first move to the first instrument after picking up the object - the placement of the object on the PEG is hesitant, and the object must be aligned in the process |  | - the first object is picked up slowly but in a directed manner - the object is transferred directly without having to move/align it - the setting down takes place directionally but with small alignment movements through the barrel tongs |  | - the first grip goes clearly and without problems to the first PEG - the sequence of object pick-up and set-down seems rehearsed and without pause - no movement is too long and always has a clear goal - The placement takes place without alignment of the object |

1. Bimanual Handling

| 1 | 2 | 3 | 4 | 5 |
| --- | --- | --- | --- | --- |
| - the pickup is done only by one instrument, and the other instrument is left at the other end of the screen - no transfer from one instrument to the other - both instruments do not "play" with each other during transfer - both instruments stop for the transfer of the object |  | - the pickup is done by one instrument, but the other one is moving toward the first instrument in the meantime - During the transfer of the object, the instruments “play” with each other but in place - the transfer takes place in a targeted manner, whereby the transferring tongs do not move |  | - the pickup is done by one instrument, and the second instrument is already there and takes over immediately - During the transfer, the tongs “play” with each other while moving to the place of setting down - The first instrument moves back to the beginning to initiate the next pickup while the second instrument still places the object |

1. Tissue Handling

| 1 | 2 | 3 | 4 | 5 |
| --- | --- | --- | --- | --- |
| - the PEG is squeezed to the maximum - the PEG is not gripped securely and moves during transfer |  | - the object is gripped correctly and not squeezed - during the exercise, however, there seems to be tension on the object (too much interaction between the PEG and the object when setting it down, for example). |  | - the object is gripped correctly and not squeezed - during the exercise, there is no pressure on the object; it does not experience pressure when picked up from the PEG and slides relaxed on the PEG |

***Annotation Guideline - Circle Cutting***

1. Depth Perception

| 1 | 2 | 3 | 4 | 5 |
| --- | --- | --- | --- | --- |
| - the scissors approach slowly, passing the target at least once - the cuts are repeated as they are cut into the air |  | - the circle is slowly but surely reached by the grasper - the scissors’ branches are positioned correctly in more than 50% of cases - the repositioning of the grasper is slow but precise |  | - the scissors and the grasper hit the fabric in a targeted way at the beginning - repositioning of the grasper is fast and without any regrasping - the fabric is cut with each closing of the scissor |

1. Efficiency

| 1 | 2 | 3 | 4 | 5 |
| --- | --- | --- | --- | --- |
| - The circle must be gripped several times at the beginning to find the starting point - The scissors are not aligned correctly and must be corrected several times in position - Several times at the same place |  | - the circle is reached slowly but precisely, and a starting point is set and not changed - the scissors actually cut the fabric in more than 50% of cases on the line so that progress is made - The grasper is used in a supporting manner |  | - The circle is reached quickly with both instruments - the starting point is defined directly with the first cut - Each cut makes progress on the task - The instruments are moved precisely and efficiently |

1. Bimanual Handling

| 1 | 2 | 3 | 4 | 5 |
| --- | --- | --- | --- | --- |
| - the grasper starts, and the scissors are positioned later - the grasper is delayed in gripping the fabric after each cut - the instruments are changed from one hand to the other several times - the grasper is barely used to support the cutting process and/or hinders the scissors |  | - The scissors and the grasper help each other and are used tactically - The repositioning of the grasper allows the scissors to work but happens with a slight delay - Changing the instruments between hands is reasonable and not more than once - The grasper does not obstruct the scissors |  | - both instruments are moved at the same time precisely and effectively - the grasper is repositioned as soon as the scissors can no longer cut the fabric unhindered - The task is completed without changing the instruments between the hands |

1. Tissue Handling

| 1 | 2 | 3 | 4 | 5 |
| --- | --- | --- | --- | --- |
| - The first grip is rough and puts tension on the fabric - The fabric is torn, the grasping forceps tear off fibers during the change - The circle is mostly cut inaccurately - The whole pad is removed from the mount |  | - The fabric does not tear, and the fibers remain intact - Individual tensions on the fabric occur during the pulling, and the circle is nevertheless cut with only a few defects - The circle is cut mostly precisely |  | - the grasper pulls the fabric in such a way that the tension is only and exactly where the scissors want to cut - the fibers are not torn - the circle is cut precisely without leaving the marked line |

***Annotation Guideline – Gallbladder Resection***

1. Depth Perception

| 1 | 2 | 3 | 4 | 5 |
| --- | --- | --- | --- | --- |
| - The gallbladder is not grasped directly; it takes several attempts to grasp the gallbladder - The scissors are approached slowly; the cut is only made after several attempts to grasp the gallbladder - The scissors are guided to the cutting plane again and very slowly before each cut |  | - The gallbladder is grasped directly and targeted but slowly - The scissors approach slowly but precisely |  | - The gallbladder is grasped directly and quickly - The scissors are positioned precisely and with one branch each above and under the outer membrane - The cuts and repositioning of instruments are precise |

1. Efficiency

| 1 | 2 | 3 | 4 | 5 |
| --- | --- | --- | --- | --- |
| - The grip on the gallbladder has to be changed very often - The scissors have to be repositioned very often - The grasper does not support the cutting process |  | - The gallbladder is grasped < 2 times to start the procedure - The procedure seems to be fluid without pause - The grasper does support the cutting process |  | - The gallbladder is grasped once, and the outermost layer is opened with the first cut - The grasper holds up the cutting plane before each cut - The scissors cut the line on the complete length without interruption |

1. Bimanual Handling

| 1 | 2 | 3 | 4 | 5 |
| --- | --- | --- | --- | --- |
| - The first grasp on the gallbladder is done without using the second hand for help - The incisions are made without using the grasper |  | - The second hand helps to position the gallbladder but slowly - The grasper does support most of the cutting process and does barely hinder the scissors - the grasper is on the gallbladder > 50% of the time during cutting |  | - The second hand helps to position the gallbladder quickly - Each cut is made with the support of the grasper, and it does not hinder the scissors - The grasper is on the gallbladder > 90% of the time during cutting |

1. Tissue Handling

| 1 | 2 | 3 | 4 | 5 |
| --- | --- | --- | --- | --- |
| - The gallbladder is roughly grasped without careful exposure of the outer layer - When cutting the outer layer, it is cut roughly and without respect to the marked lines - During handling, the outer layer is torn open several times by tension - The gallbladder is perforated widely |  | - The gallbladder is grasped carefully with almost no tear in the outer layer - The first cut is made after tension is applied to the gallbladder so that the inner layer comes off - The incisions are mostly within the marked line - The gallbladder is not perforated |  | - The outer layer is safely and visibly separated from the inner layer by careful tension by the grasper - The first cut is very fine and minimally opens the gallbladder - All cuts are within the marked line - The gallbladder is not perforated |

***Annotation Guideline - Laparoscopic Surgical Knot***

1. Depth Perception

| 1 | 2 | 3 | 4 | 5 |
| --- | --- | --- | --- | --- |
| - Grasping the penrose drain/the needle/the thread needs several attempts - The needle is brought very slowly only to the drainage; the target is not pricked |  | - The needle is picked up and orientated in one attempt but slowly - The needle is brought very slowly toward the drainage - The thread is wrapped around the needle holder slowly but without errors |  | - The needle is picked up and orientated in one attempt and quickly - The needle is brought precisely and quickly toward the drainage - The thread is wrapped around the needle holder quickly and without errors |

1. Efficiency

| 1 | 2 | 3 | 4 | 5 |
| --- | --- | --- | --- | --- |
| - The alignment of the needle before stitching is bad so that realignment is needed - The alignment of the needle for knotting is bad so that knotting is hindered |  | - The needle is clamped correctly after some positioning - The stitching is slow; however, the participant progresses in the exercise - Knotting is possible but slow |  | - The needle alignment is correct on the first try - The stitch in and out of the penrose drain can be done without correction in one sweep - The grasper directly takes over the needle, and the first knot can directly be placed |

1. Bimanual Handling

| 1 | 2 | 3 | 4 | 5 |
| --- | --- | --- | --- | --- |
| - The alignment of the needle is performed only with the grasper/only with the needle holder - The grasper does not grab the penrose drain for better visualization of the marked spots - The needle holder pushes the needle through the penrose and takes it off again; the grasper is not used at all - The instruments hinder each other |  | - When aligning the needle, one instrument mainly takes over the alignment; the second one helps only occasionally - The grasper does grab the penrose drain, but hinders the needle holder - When piercing, first the movement of the needle holder is finished, then the grasping forceps take over - Mostly only one instrument moves during the knotting process |  | - Both instruments help to align the needle - The grasper does grab the penrose drain to show the marked spots - When inserting the needle, the needle holder turns the needle thus far that the grasping forceps can take over directly - When wrapping the thread around the needle holder, both instruments move together - The transfer/takeover is always done by two active instruments |

1. Tissue Handling

| 1 | 2 | 3 | 4 | 5 |
| --- | --- | --- | --- | --- |
| - The needle is bent/suture is ripped or shows strong tension during initial adjustment - The needle is pulled straight through the penrose drain and is not turned in a semicircle - The suture is pulled through the penrose drain in a way that cuts the drain - The penrose drain is pulled off the surface |  | - The needle is adjusted with little force and/or little tension on the threat - The penrose is brought to tension for a short time, but only small tearing of the tissue can be seen - Overall, the knotting is "forced", but the knot is loose |  | - The needle is adjusted in a relaxed manner without bending or tension on the thread - The drainage experiences minimal tension and no tearing during the stitch and the pulling of the thread - the knot is put down without tearing or excessive tension and is tight |
